# Dissociation of μ- and δ-opioid inhibition of glutamatergic synaptic transmission in superficial dorsal horn

**DOI:** 10.1186/1744-8069-6-71

**Published:** 2010-10-26

**Authors:** Paul J Wrigley, Hyo-Jin Jeong, Christopher W Vaughan

**Affiliations:** 1 Pain Management Research Institute, Kolling Institute for Medical Research, Northern Clinical School, The University of Sydney at Royal North Shore Hospital, St Leonards, NSW 2065, Australia

## Abstract

**Background:**

There is anatomical and behavioural evidence that μ- and δ-opioid receptors modulate distinct nociceptive modalities within the superficial dorsal horn. The aim of the present study was to examine whether μ- and δ-opioid receptor activation differentially modulates TRP sensitive inputs to neurons within the superficial dorsal horn. To do this, whole cell patch clamp recordings were made from lamina I - II neurons in rat spinal cord slices *in vitro *to examine the effect of opioids on TRP agonist-enhanced glutamatergic spontaneous miniature excitatory postsynaptic currents (EPSCs).

**Results:**

Under basal conditions the μ-opioid agonist DAMGO (3 μM) reduced the rate of miniature EPSCs in 68% of neurons, while the δ- and κ-opioid agonists deltorphin-II (300 nM) and U69593 (300 nM) did so in 13 - 17% of neurons tested. The TRP agonists menthol (400 μM) and icilin (100 μM) both produced a Ca^2+^-dependent increase in miniature EPSC rate which was unaffected by the voltage dependent calcium channel (VDCC) blocker Cd^2+^. The proportion of neurons in which deltorphin-II reduced the miniature EPSC rate was enhanced in the presence of icilin (83%), but not menthol (0%). By contrast, the proportion of DAMGO and U69593 responders was unaltered in the presence of menthol (57%, 0%), or icilin (57%, 17%).

**Conclusions:**

These findings demonstrate that δ-opioid receptor activation selectively inhibits inputs activated by icilin, whereas μ-opioid receptor activation has a more widespread effect on synaptic inputs to neurons in the superficial dorsal horn. These findings suggest that δ-opioids may provide a novel analgesic approach for specific, TRPA1-like mediated pain modalities.

## Background

The superficial dorsal horn of the spinal cord is a major site of μ-opioid receptor mediated analgesia [1]. There is some evidence that spinally delivered δ-opioid agonists also inhibit behavioural responses to a range of noxious stimuli [2-9]. It has been suggested, however, that μ-opioid receptors mediate the analgesic actions of δ-opioid agonists, particularly at high doses [e.g. [10,11]]. Alternatively, the variability in the analgesic efficacy of spinally delivered δ-opioid agonists might be due to the use of different pain assays in these studies [12,13]. In this regard, it has recently been demonstrated that noxious thermal and mechanical pain are mediated by distinct primary afferents and spinal pathways, and that these pathways are differentially modulated by μ- and δ-opioid receptors [13,14].

Opioids are thought to produce their effects within the dorsal horn by inhibiting neurotransmitter release from the central terminals of primary afferent nociceptors, and by postsynaptic inhibition of dorsal horn neurons [1]. A number of *in vitro *studies have reported that μ-opioid receptor agonists presynaptically inhibit glutamatergic synaptic transmission onto subpopulations of lumbar and trigeminal dorsal horn neurons [15-21]. In these studies μ-opioids inhibit afferent evoked glutamatergic excitatory postsynaptic currents (EPSCs) and reduce the rate of spontaneous miniature EPSCs. By contrast, there are mixed reports on δ-opioid receptor agonists, with some studies demonstrating inhibition of glutamatergic synaptic transmission in dorsal horn neurons [18,20,21] and others reporting that they have little, or no effect [15,17,19].

The examination of opioid actions on specific nociceptive afferent inputs to dorsal horn neurons is limited with the *in vitro *approach because of the inability to naturally activate different afferent modalities, although this may be overcome with the *in vivo *patch clamping approach [22-24]. The transport of a range of proteins, including transient receptor potential (TRP) ion channels, to the central terminals of primary afferent fibres provides a means to pharmacologically differentiate between subpopulations of nociceptive afferent inputs to dorsal horn neurons in an *in vitro *preparation. This approach has been used previously to differentiate TRPV1, TRPM8 and TRPA1 sensitive inputs to dorsal horn neurons in intact spinal cord slices [25-32]. In these studies, TRP-mediated presynaptic enhancement of glutamatergic synaptic transmission is observed as an increase in miniature EPSC rate, although, TRP agonists produce a paradoxical decrease in afferent evoked EPSCs [but see [33]]. In the present study we used an *in vitro *approach to examine whether different opioid receptor subtypes modulate specific TRP-sensitive inputs to neurons within the superficial dorsal horn by examining their opposing effects on spontaneous miniature EPSCs.

## Methods

### Animals

Experiments were carried out on male Sprague-Dawley rats (14 - 21 days old), following the guidelines of the 'NH&MRC Code of Practice for the Care and Use of Animals in Research in Australia' and with the approval of the Royal North Shore Hospital Animal Care and Ethics Committee. Animals were anaesthetized with halothane (1 - 3% in O_2_) and a laminectomy was performed to expose the lumbar spinal cord. The dura was incised and the spinal column quickly removed and placed in ice cold artificial cerebrospinal fluid (ACSF) of composition: (mM): NaCl, 126; KCl, 2.5; NaH_2_PO_4_, 1.4; MgCl_2_, 1.2; CaCl_2_, 2.4; glucose, 11; NaHCO_3_, 25. The animal was then euthanased by decapitation. Transverse (300 μm) slices of the lumbar spinal cord (L4 - 6) were cut and maintained at room temperature in a submerged chamber containing ACSF equilibrated with 95% O_2 _and 5% CO_2_. The slices were then transferred to a chamber and superfused continuously (1.8 ml.min^-1^) with ACSF at 34°C using an in-line temperature controller (CL-100, Warner Instruments, Hampden, USA). In some experiments ACSF was modified. NaH_2_PO_4 _was omitted in Cd^2+ ^experiments; Mg^2+ ^was increased to 10 mM in Ca^2+^-free experiments; NaCl was reduced to 103.5 mM in high K^+ ^(KCl 25 mM) experiments. In these cases, the altered ACSF was superfused onto the slice for at least 30 min before recordings were commenced.

### Electrophysiology

Dorsal horn neurons were visualized using infra-red Nomarski, or Dodt-tube optics on an upright microscope (Olympus BX50, Olympus, Sydney, Australia). Whole-cell voltage clamp recordings (holding potential -65 mV, liquid junction potential corrected) were made using an Axopatch 200B amplifier (Molecular Devices, Sunnyvale, CA, USA) with patch clamp electrodes (2 - 5 MΩ). The internal solution contained (mM): CsMeSO_3 _135, EGTA 10, HEPES 5, NaCl 10, MgCl_2 _1 and MgATP 2. All internal solutions were adjusted to pH 7.3 and osmolality 280 - 285 mosmol.l^-1^. Series resistance (< 25 MΩ) was compensated by 80% and continuously monitored during experiments.

Spontaneous miniature EPSCs were recorded in the presence of tetrodotoxin (TTX, 500 nM), the GABA_A _channel blocker picrotoxin (100 μM) and the glycine receptor antagonist strychnine (3 μM). EPSCs were filtered (2 kHz low-pass filter) and sampled (10 kHz) in 4 second epochs every 6 seconds for analysis using AxographX (Axograph Scientific, Sydney, Australia). For analysis, miniature EPSCs above a preset threshold (4.0 - 4.5 standard deviations above baseline noise) were automatically detected by a sliding template algorithm and then manually checked. We did not exclude, or separate merged EPSCs (with < 5 ms inter-event interval separation) which sometimes occurred at high EPSC rates and led to an increase in the mean EPSC amplitude. Neurons were considered to be opioid responders if there was a decrease in miniature EPSC rate which was greater than 15% and returned to baseline after washout, or addition of an antagonist.

### Chemicals and statistical analysis

Allyl-isothiocyanate, CTAP (D-Phe-Cys-Tyr-D-Trp-Arg-Pen-Thr-NH_2_), DAMGO (Tyr-D-Ala-Gly-N-Me-Phe-Gly-ol enkephalin), deltorphin-II, (1*R*,2*S*,5*R)-*(-)-menthol, picrotoxin, strychnine hydrochloride and U69593 were obtained from Sigma (Sydney, Australia); icilin (3,6-dihyrdo-1-(2-hydroxyphenyl)-4-(3-nitrophenyl)-2(1H)-pyrimidinone) was from Cayman Chemical Co. (Ann Arbor, U.S.A.); (E)-capsaicin, CNQX (6-cyano-7-nitroquinoxaline-2,3-dione disodium), ICI-174,864, naloxone and nor-BNI (nor-binaltorphimine dihydrochloride) were from Tocris Cookson (Bristol, U.K.); QX 314 and TTX (tetrodotoxin) were from Alomone (Jerusalem, Israel). Stock solutions of all drugs were made in distilled water, except U69593 (in 0.1 M HCl), icilin (in dimethylsulfoxide), capsaicin and menthol (in ethanol). These were diluted to working concentrations using ACSF immediately before use, except for picrotoxin which was dissolved directly into ACSF, and were applied by superfusion. The order in which opioid agonists were applied was randomised. All statistical comparisons were made using one- and two-way ANOVAs, chi-squared and Student's paired/un-paired t-tests. All pooled data are expressed as mean ± S.E.M.

## Results

### Opioid inhibition of miniature EPSCs under basal conditions is largely mediated by μ-opioid receptors

**Figure 1:**
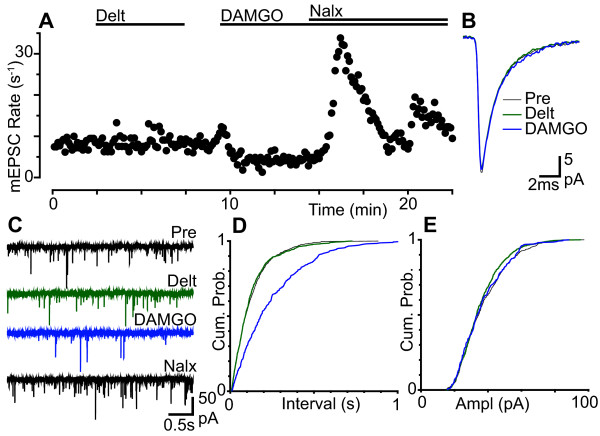
**μ-Opioids presynaptically inhibit basal glutamatergic transmission in superficial dorsal horn neurons**. (a) Time plot of miniature EPSC (mEPSC) rate during superfusion of deltorphin-II (Delt, 300 nM), DAMGO (3 μM) and naloxone (Nalx, 1 μM). (b) Averaged traces and (c) raw current traces of mEPSCs prior to (Pre), and during deltorphin-II, DAMGO and naloxone. Cumulative probability distribution plots of mEPSC (d) inter-event interval and (e) amplitude for the epochs averaged in (b) (number of events = 635, 640, 327 for control, deltorphin-II and DAMGO, respectively, over 80 s intervals). (a) - (e) are taken from one neuron.

**Figure 2:**
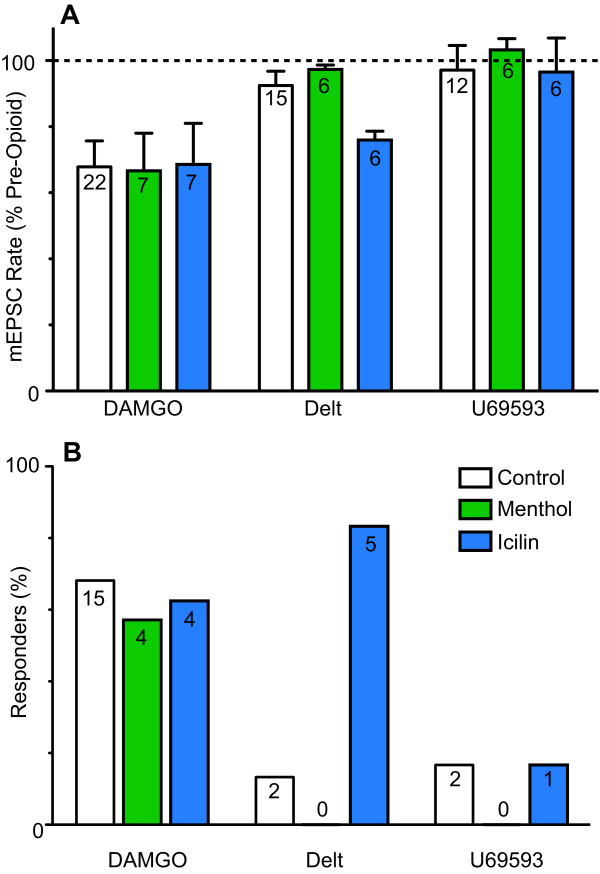
**Effect of opioids on basal, and menthol and icilin enhanced miniature EPSCs**. (a) Bar chart showing the percentage inhibition of miniature EPSC (mEPSC) rate produced by DAMGO (3 μM), deltorphin-II (Delt, 300 nM) and U69593 (300 nM), expressed as a percentage of the pre-opioid level and averaged across all neurons tested. (b) Bar chart showing the percentage of neurons in which DAMGO, deltorphin-II and U69593 produced a reduction in mEPSC rate of greater than 15%. Data in (a) and (b) are shown for neurons in which opioids were superfused alone, or in the additional presence of menthol (400 μM), or icilin (100 μM). The number in the bars represent (a) the total number of neurons tested and (b) the number of neurons which responded to each opioid.

We first examined the effect of μ-, δ- and κ-opioid receptor activation on miniature EPSCs in lamina I - II neurons of the dorsal horn under basal conditions. In the presence of TTX (500 nM), picrotoxin (100 μM) and strychnine (3 μM), superfusion of the μ-opioid receptor agonist DAMGO (3 μM) produced a decrease in the rate of miniature EPSCs in 68% (n = 15/22) of neurons tested (Figure 1a, c, 2b). In the responding neurons, the miniature EPSC rate during superfusion of DAMGO was 51 ± 5% of the pre-opioid level (n = 15). When averaged across all neurons, the miniature EPSC rate during superfusion of DAMGO was 68 ± 8% of the pre-opioid level (Figure 2a, p = 0.0005, n = 22). The decrease in miniature EPSC rate produced by DAMGO was reversed by the addition of the μ-opioid receptor antagonist CTAP (1 μM, n = 3), or the non-selective opioid receptor naloxone (1 μM, n = 10) which was in some cases associated with rebound facilitation (Figure 1a, c). The decrease in miniature EPSC rate produced by DAMGO was associated with a rightward shift in the cumulative probability distributions of miniature EPSC inter-event intervals (Figure 1d). DAMGO had no effect on the kinetics and amplitude of miniature EPSCs, or on the cumulative probability distributions of miniature EPSC amplitudes (Figure 1b, e).

The δ-opioid receptor agonist deltorphin-II (300 nM) had no effect on miniature EPSCs in most neurons (Figure 1). Deltorphin II reduced the rate of miniature EPSCs in only 13% (n = 2/15) of neurons tested (Figure 2b; 47 and 79% of pre-opioid level in the responders). When averaged across all neurons, the miniature EPSC rate during superfusion of deltorphin-II was 95 ± 6% of the pre-opioid level (Figure 2a, p = 0.4, n = 15). In the deltorphin-II responding neurons, the decrease in miniature EPSC rate was reversed by the addition of the δ-opioid receptor antagonist ICI-174864 (1 μM, n = 2). It might be noted however that a higher concentration of deltorphin (10 μM) produced a decrease in miniature EPSC rate in 71% of neurons tested (n = 5/7), which was significant when averaged across all neurons (78 ± 7% of the pre-opioid level, p = 0.02, n = 7). This high concentration deltorphin-II (10 µM) induced inhibition was reversed by addition of ICI-174864 (1 μM) in only one neuron and was reversed by addition of CTAP (1 μM) in the other four neurons. Deltorphin-II, at both concentrations, had no effect on the kinetics and amplitude of miniature EPSCs, or on the cumulative probability distributions of miniature EPSC amplitudes (Figure 1b, e).

Finally, the κ-opioid receptor agonist U69593 (300 nM) reduced the miniature EPSC rate in only 17% (n = 2/12) of neurons tested (Figure 2b; 50 and 75% of pre-opioid level in the two responders). When averaged across all neurons, the miniature EPSC rate during superfusion of U69593 was 98 ± 6% of the pre-opioid levels (Figure 2a, p = 0.7, n = 12). In the U69593 responding neurons, the decrease in miniature EPSC rate was reversed by the addition of the κ-opioid receptor antagonist nor-BNI (300 nM, n = 2). U69593 had no effect on the kinetics and amplitude of miniature EPSCs, or on the cumulative probability distributions of miniature EPSC amplitudes. Overall, these observations suggest that μ-opioid receptor activation presynaptically inhibits basal glutamatergic synaptic transmission in a much larger subpopulation of superficial dorsal horn neurons, than δ-, or κ-opioid receptor activation.

### Opioid inhibition of miniature EPSCs is unaffected by menthol

**Figure 3:**
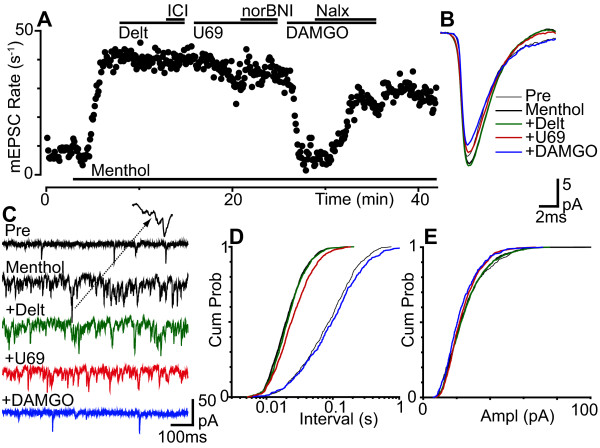
**Presynaptic μ-opioid inhibition predominates during menthol enhanced glutamatergic transmission**. (a) Time plot of miniature EPSC (mEPSC) rate during superfusion of menthol (400 μM), then during addition of deltorphin-II (Delt, 300 nM), ICI-174864 (ICI, 1 μM), U69593 (U69, 300 nM), nor-BNI (300 nM), DAMGO (3 μM) and naloxone (Nalx, 1 μM). (b) Averaged traces and (c) raw current traces of mEPSCs prior to (Pre), and during menthol, then during addition of deltorphin-II, U69593 and DAMGO. Inset in (c) is an expanded part of the trace to show summation of mEPSCs in the presence of menthol. Cumulative probability distribution plots of mEPSC (d) inter-event interval and (e) amplitude for the epochs averaged in (b) (number of events = 648, 1597, 1540, 1259, 506 for control, menthol, deltorphin-II, U69593 and DAMGO over 80, 40, 40, 40 and 80 s intervals, respectively). (a) - (e) are taken from one neuron.

Superfusion of menthol (400 μM) produced an increase in miniature EPSC rate in 86% of neurons tested (Figure 3a, c, n = 12/14). On average the miniature EPSC rate was 7.9 ± 1.8 s^-1 ^and 25.3 ± 3.4 s^-1 ^prior to, and during application of menthol (p = 0.002, n = 14). The increase in miniature EPSC rate produced by menthol was associated with a leftward shift in the cumulative probability distribution of the miniature EPSC inter-event intervals (Figure 3d). In many of the responding neurons (n = 8/12), menthol also produced an increase in the amplitude of miniature EPSCs which was due to summation of individual events during burst-like activity (Figure 3b, c, 133 ± 9% of pre-menthol, p = 0.005, n = 14).

We next examined the actions of opioids in neurons which responded to menthol with an increase in miniature EPSC rate. In the presence of menthol (400 μM), DAMGO (3 μM) produced a decrease in the rate of miniature EPSCs in 57% (n = 4/7) of neurons tested which was reversed by the addition of CTAP (1 μM, n = 2), or naloxone (1 μM, n = 2) (Figure 2b, 3a, c). Under these conditions, the miniature EPSC rate during superfusion of DAMGO in the responding neurons was 45 ± 9% of pre-opioid levels (n = 4). By contrast, deltorphin-II (300 nM, n = 6) and U69593 (300 nM, n = 6) did not produce a decrease in the rate of miniature EPSCs in any neurons tested in the presence of menthol (Figure 2b, 3a, c). When averaged across all neurons, the miniature EPSC rate during superfusion of DAMGO, deltorphin-II and U69593 was 67 ± 11% (n = 7), 97 ± 1% (n = 6) and 103 ± 3% (n = 6) of pre-opioid levels, respectively (Figure 2a, p = 0.03, 0.1, 0.4). DAMGO, but not deltorphin-II and U69593 produced a rightward shift in the cumulative probability distribution of the miniature EPSC inter-event intervals (Figure 3d). On average, DAMGO, deltorphin-II and U69593 had no effect on the kinetics and amplitude of miniature EPSCs, or on the cumulative probability distributions of miniature EPSC amplitudes in the presence of menthol (Figure 3b, e).

### High micromolar icilin increases the incidence of δ-opioid inhibition of miniature EPSCs

**Figure 4:**
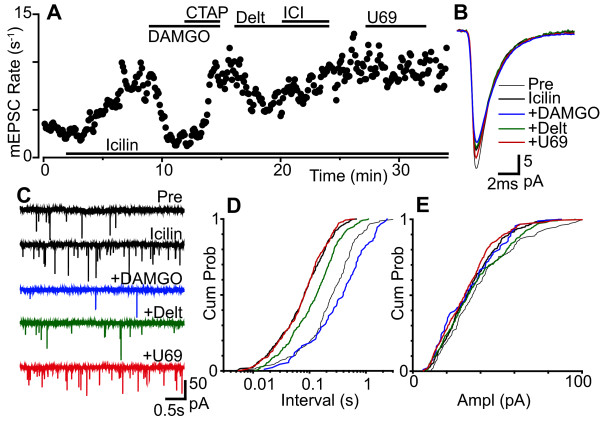
**Both μ- and δ-opioids inhibit icilin enhanced glutamatergic transmission**. (a) Time plot of miniature EPSC (mEPSC) rate during superfusion of icilin (100 μM), then during addition of DAMGO (3 μM), CTAP (1 μM), deltorphin-II (Delt, 300 nM), ICI-174864 (ICI, 1 μM) and U69593 (U69, 300 nM). (b) Averaged traces and (c) raw current traces of mEPSCs prior to (Pre), and during icilin, then during addition of deltorphin-II, U69593 and DAMGO. Cumulative probability distribution plots of mEPSC (d) inter-event interval and (e) amplitude for the epochs averaged in (b) (number of events = 155, 372, 91, 343, 438 for control, icilin, DAMGO, deltorphin-II and U69593 over 60, 44, 52, 64 and 48 s intervals, respectively). (a) - (e) are taken from one neuron.

Superfusion of icilin (100 μM) produced an increase in miniature EPSC rate in 79% of neurons tested (Figure 4a, c, n = 11/14). On average the miniature EPSC rate was 5.6 ± 0.5 s^-1 ^and 19.9 ± 2.7 s^-1 ^prior to, and during application of icilin (p = 0.0004, n = 14). The increase in miniature EPSC rate produced by icilin was associated with a leftward shift in the cumulative probability distribution of the miniature EPSC inter-event intervals (Figure 4d). In some of the responding neurons (n = 4/11), icilin also produced an increase in the amplitude of miniature EPSCs which was due to summation of individual events during burst-like activity (128 ± 9% of pre-icilin, p = 0.01, n = 11).

We next examined the actions of opioids in neurons which responded to icilin with an increase in miniature EPSC rate. In the presence of icilin (100 μM), DAMGO (3 μM) and deltorphin-II (300 nM) produced a decrease in the rate of miniature EPSCs in 57% (n = 4/7) and 83% (n = 5/6) of neurons tested (Figure 2b, 4a, c). By contrast, U69593 (300 nM) produced a decrease in the rate of miniature EPSCs in only 17% (n = 1/6) of neurons tested in the presence of icilin (Figure 2b, 4a, c). Under these conditions, the miniature EPSC rate during superfusion of DAMGO, deltorphin-II and U69593 in the responding neurons was 44 ± 9%, 74 ± 2% and 47% of pre-opioid levels, respectively (n = 4, 7, 1). When averaged across all neurons, the average miniature EPSC rate during superfusion of DAMGO, deltorphin-II and U69593 was 69 ± 12%, 76 ± 3% and 97 ± 10% of pre-opioid levels, respectively (Figure 2a, p = 0.04, 0.0003, 0.8, n = 7, 6, 6). The decrease in miniature EPSC rate produced by DAMGO, deltorphin-II and U69593 was reversed by the addition of CTAP (1 μM, n = 4), ICI-174864 (1 μM, n = 5) and nor-BNI (300 nM, n = 1), respectively (Figure 4a). The decrease in miniature EPSC rate produced by DAMGO, deltorphin-II and U69593 was associated with a rightward shift in the cumulative probability distribution of the miniature EPSC inter-event intervals (Figure 4d). On average, DAMGO, deltorphin-II and U69593 had no effect on the kinetics and amplitude of miniature EPSCs, or on the cumulative probability distributions of miniature EPSC amplitudes in the presence of icilin (Figure 4b, e).

### Role of presynaptic calcium

We next examined whether the difference in δ-opioid modulation of menthol and icilin enhanced synaptic transmission was related to presynaptic calcium mechanisms. To do this, we compared the increase in miniature EPSC rate produced by icilin and menthol under control conditions (normal ACSF), to that in the presence of the voltage dependent calcium channel (VDCC) blocker Cd^2+ ^(30 μM), or in the absence of external Ca^2+^. The basal miniature EPSC rate was less in the presence of Cd^2+ ^(1.7 ± 0.4 s^-1^, p < 0.001, n = 16) and in the absence of external Ca^2+ ^(3.3 ± 0.5 s^-1^, p < 0.01, n = 21), compared to that in normal ACSF (6.6 ± 0.9 s^-1^, n = 38). The basal miniature EPSC amplitude was similar in the presence of Cd^2+^, in the absence of external Ca^2+ ^and in normal ACSF (32 ± 4 pA, 31 ± 2 pA and 28 ± 2 pA, p = 0.3).

**Figure 5:**
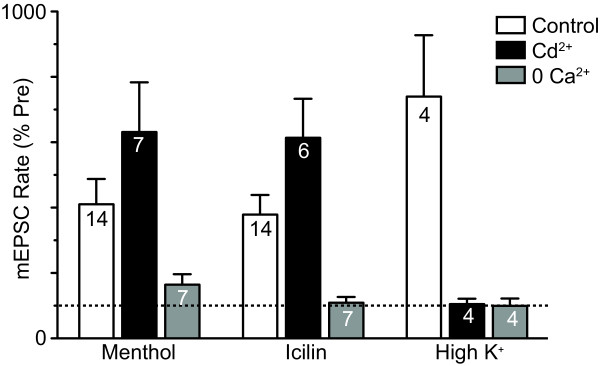
**Menthol and icilin enhancement of glutamatergic transmission is independent of voltage dependent calcium channels**. Bar chart showing the change in miniature EPSC (mEPSC) rate produced by menthol (400 μM), icilin (100 μM) and elevated external K^+ ^(25 mM), in normal ACSF (control), in ACSF containing Cd^2+ ^(30 μM), and in Ca^2+^-free ACSF (0 Ca^2+^). Data are expressed as a percentage of the pre-menthol, icilin and high K^+ ^levels, and averaged across all neurons. The number in the bars represent the total number of neurons tested under each condition.

In the presence of Cd^2+ ^(30 μM), menthol (400 μM) produced an increase in the rate of miniature EPSCs (p = 0.009, n = 7) which was not significantly different to that produced in normal ACSF (n = 14) (Figure 5, p = 0.07). Icilin (100 μM) also produced an increase in the rate of miniature EPSCs in the presence of Cd^2+ ^(p = 0.03, n = 6) which was not significantly different to that produced in its absence (n = 14) (Figure 5, p = 0.1). By contrast, menthol and icilin did not produce a significant increase in miniature EPSC rate in the absence of external Ca^2+^(Figure 5, p = 0.09, 0.6, n = 7, 7). As a control we also examined whether raising the external concentration of potassium produced a calcium dependent increase in miniature EPSC rate. In all neurons tested, increasing external potassium ([K^+^]_ext _= 25 mM) produced an increase in the rate of miniature EPSCs in normal ACSF (p = 0.04, n = 4), but not in the presence of Cd^2+ ^(30 μM, p = 0.8, n = 4), or in the absence of external Ca^2+^(p = 0.9, n = 4) (Figure 5).

## Discussion

The present findings suggest that δ-opioids presynaptically inhibit a distinct subpopulation of glutamatergic inputs onto dorsal horn neurons which are activated by the TRP agonist icilin. By contrast, activation of presynaptic μ-opioid receptors inhibits multiple types of glutamatergic inputs onto dorsal horn neurons. These findings provide cellular evidence that μ- and δ-opioid receptors are expressed by partially distinct inputs to dorsal horn neurons and potentially subserve different pain modalities.

In the present study the inhibition of glutamatergic synaptic transmission produced by low micromolar concentrations of DAMGO, deltorphin-II and U69593 was mediated via μ-, δ- and κ-opioid receptors. This inhibition was likely to be presynaptic because opioids produced a reduction in the rate of asynchronous spontaneous miniature EPSCs, without any change in their amplitude, or kinetics. Our findings are largely consistent with previous reports of μ-opioid receptor mediated presynaptic inhibition of glutamatergic synaptic transmission in the majority of lamina I - II superficial dorsal horn neurons under basal conditions [15-20]. The low incidence of δ- and κ-opioid receptor mediated inhibition reflects the mixed findings of previous studies, in which δ- and κ-opioid agonists have been reported to either inhibit, or have little, or no effect on glutamatergic synaptic transmission in dorsal horn neurons [15,17-20,34,35]. The differences in the proportions of opioid responders between these studies may have been due to the use of different response criterion (e.g. responders if inhibition > 15% in the present study versus 5% in Kohno et al. [20]). The differences may have also been due to the use of high agonist concentrations in some *in vivo *and *in vitro *studies [17,18,21,34] and the possible involvement of μ-opioid receptors in the actions of δ-opioid agonists [e.g. [11]]. Indeed, we found that higher concentrations of deltorphin-II (10 μM) produced both μ- and δ-opioid receptor mediated presynaptic inhibition. It is also possible that these differences were due to variations in presynaptic excitability, VDCCs and intracellular calcium buffering (see below).

The enhancement of glutamatergic synaptic transmission by menthol and icilin was likely to be due to a presynaptic action because they produced an increase in the rate of miniature EPSCs, as shown previously for a range of TRP agonists [25-29,31-33]. The presynaptic actions of menthol and icilin are partly consistent with activation of TRPM8 and TRPA1, respectively [36-38]. While menthol is typically used as a TRPM8 agonist, it has also been reported to be a ligand at TRPA1 and TRPV3 ion channels [39,40]. In addition, icilin activates TRPM8 and TRPA1 at low and high micromolar concentrations, respectively [32,36,38]. The actions of high micromolar icilin in the present study are more consistent with TRPA1 activation because the enhancement of spinal glutamatergic synaptic transmission by high micromolar icilin is highly correlated to that produced by activation of TRPA1, but not TRPM8 [[29], see also [32]]. We did not confirm the identity of the TRP channels with other TRPA1 agonists, such as allyl-isothiocyanate and cinnamaldehyde, because they produce rapidly desensitising increases in miniature EPSC rate [29] that precluded subsequent examination of opioid inhibition. Thus, the precise role of TRPM8 and TRPA1 in the presynaptic enhancement of synaptic transmission produced by both menthol and icilin remains to be confirmed.

The menthol and icilin induced enhancement of synaptic transmission and the subsequent opioid inhibition were likely to be due to an action on the terminals of primary afferents, as opposed to the terminals of spinal interneurons, or descending medullo-spinal fibres. There is anatomical evidence that TRPM8 and TRPA1 are expressed exclusively by dorsal root ganglion (DRG) neurons and their central terminals [36-38,41]. In addition, slice studies have shown that, like capsaicin, menthol, icilin and cinnamaldehyde inhibit dorsal root evoked EPSCs, with TRPA1 agonists specifically inhibiting C-fibre evoked EPSCs [[29-31], but see [33]]. This paradoxical TRP induced inhibition of evoked EPSCs precluded the examination of opioid inhibition of menthol and icilin sensitive primary afferent evoked EPSCs. As a result, in the present study miniature EPSCs, rather than primary afferent evoked EPSCs were examined.

μ-Opioid receptor activation inhibited basal, and menthol/icilin-enhanced glutamatergic synaptic transmission in 57 - 68% of neurons tested. By contrast, δ-opioid receptor activation inhibited icilin-enhanced glutamatergic synaptic transmission in virtually all neurons tested, but had little effect on basal and menthol-enhanced glutamatergic synaptic transmission. The difference in μ- and δ-opioid inhibition may have been due to variations in presynaptic opioid signalling. It has previously been reported that μ-opioid inhibition of miniature EPSCs in lamina I is mediated by presynaptic VDCCs [15]. While μ-opioid receptor activation inhibits VDCCs in subpopulations of DRG neurons, the observation of δ- and κ-opioid inhibition varies between studies [21,42-46]. We found that the increase in miniature EPSC rate produced by both menthol and icilin was dependent upon external calcium, but was unaffected by presynaptic VDCC blockade, as observed previously for menthol, allyl-isothiocyanate and cinnamaldehyde [28,31,33]. The μ- and δ-opioid inhibition observed in the presence of menthol and icilin was therefore not likely to be mediated by presynaptic VDCCs and cannot account for the observed differences. The dependence upon external calcium is consistent with the finding that TRPM8 enhancement of synaptic transmission is mediated by release of calcium from intracellular stores within afferent nerve terminals [33]. While it is interesting to note that intracellular calcium regulation varies between different types of DRG neurons [47], there are few reports of opioid regulation of intracellular calcium [48,49]. The precise mechanisms underlying opioid modulation of TRPM8 and TRPA1 enhanced synaptic transmission therefore remain to be determined.

The difference in μ- and δ-opioid inhibition may also have been due to δ-opioid receptor translocation. There is evidence to suggest that unlike μ-opioid receptors, δ-opioid receptors are largely located in intracellular compartments of nerve terminals in DRG neurons and the dorsal horn under normal conditions [50-54]. Stimuli, such as depolarisation, swim-stress, inflammation, chronic morphine treatment, TRPV1 and δ-opioid receptor activation, induce translocation of δ-opioid receptors to the surface membrane within DRG neurons, the dorsal horn and brain [49,54-58]. In this regard, electrophysiological studies have reported *de novo *δ-opioid inhibition of synaptic transmission in descending analgesic pathways following chronic morphine treatment [59-62]. Simple activity-dependent receptor externalisation, however, does not readily explain the increased δ-opioid sensitivity observed in the presence of icilin because similar increases in miniature EPSC rate were seen with both menthol and icilin.

The most likely explanation for the observed differential opioid inhibition is that δ-opioids act on a distinct subpopulation of afferent nerve terminals within the superficial dorsal horn which express TRPA1-like ion channels. Anatomical studies have shown that TRP channel expression in DRG neurons and their central terminals is heterogeneous, with TRPA1 being expressed in TRPV1-containing, peptidergic C-fibre afferents and TRPM8 being expressed by a diverse range of Aδ- and C-fibre afferents [36-38,41,63]. This is consistent with electrophysiological evidence that putative TRPV1, TRPM8 and TRPA1 agonists target partially distinct presynaptic inputs to subpopulations of dorsal horn neurons [28,29,31,32]. While there is considerable evidence for a substantial overlap in the expression of μ- and δ-opioid receptors in DRG neurons [e.g. [8,21,64]] it has recently been suggested that μ- and δ-opioid receptors are expressed in distinct subpopulations of peptidergic and non-peptidergic DRG neurons, respectively [13,14]. The latter does not entirely match our suggestion of δ-opioid receptor/TRPA1 co-expression, although the precise relationship between TRP ion channel and opioid receptor expression in DRG neurons remains to be determined. It is interesting to note that μ- and δ-opioid receptor containing afferent pathways have been reported to convey noxious thermal and mechanical nociception, respectively [13]. In addition, TRPA1 has been reported to respond to noxious cold, mechanical pressure, exogenous and endogenous irritants, and has been implicated in chronic pain states [for review see [65,66]]. While little is known about the clinical utility of δ-opioids [67], the present findings suggest that they may have potential in specific pain modalities, including noxious mechanical forces and irritants, and in chronic pain states.

## Conclusions

The present results suggest that, unlike μ-opioid receptors, δ-opioid receptor activation presynaptically inhibits a specific subpopulation of excitatory glutamatergic inputs onto dorsal horn neurons. This δ-opioid sensitive subpopulation of inputs could potentially correlate to the recently described afferent pathway which conveys noxious mechanical sensation. In addition, our findings suggest that the δ-opioid sensitive inputs convey TRPA1-like noxious chemical sensation, although a role for other icilin-sensitive TRP channels cannot be excluded. Together, these findings suggest that δ-opioid agonists may provide a novel analgesic target for specific pain modalities.

## Competing interests

The authors declare that they have no competing interests.

## Authors' contributions

The study was conceived and the experiments were designed by PJW and CWV. PJW, HJJ and CWV performed the patch clamp experiments and analysed electrophysiological data. PJW made the figures. All authors contributed to writing the manuscript, and all read and approved the final manuscript.
